# Book Review: Age-Friendly Cities and Communities in International Comparison: Political Lessons, Scientific Avenues, and Democratic Issues

**DOI:** 10.3389/fsoc.2019.00002

**Published:** 2019-01-28

**Authors:** Agnieszka Cieśla

**Affiliations:** Department for Spatial Planning and Environmental Sciences, Warsaw University of Technology, Warsaw, Poland

**Keywords:** aging, age-friendly cities and communities, active aging, healthy aging, book review

The world's population is rapidly aging. This process poses great challenges particularly for cities and communities, which have to reshape their development policies that so far have been focused on younger population groups. One of the answers to these challenges on a global scale is the initiative of the Global Network of Age-Friendly Cities and Communities (GNAFCC) founded in 2010 by the World Health Organization. The network is expanding at a high rate. Initially, it consisted of 33 cities, while when writing these words (at the end of the year 2018) the network has already expanded to over 700 cities and communities worldwide.

Among numerous publications on the topic, one of the first that thoroughly discusses this initiative was “Age-Friendly Cities and Communities in International Comparison: Political Lessons, Scientific Avenues and Democratic Issues” published in 2016 and edited by Thibauld Moulaert and Suzanne Garon with contributions by a number of renowned experts and scholars. According to editors, the book objective is to investigate the origins of the AFCC movement, present its development worldwide and focus on future challenges. The book is divided into three parts and 17 papers. The structure is well reflecting the purpose.

The first part is about the origins and contemporary developments of AFCC in the light of the active aging concept. A critical remark was made by the editors who pointed out that researchers are not just experts anymore but tend to be mobilized actors, whose engagement in the implementation of AFCC initiatives is indispensable. This is supported by the attitude of Alexandre Kalache who is not only a key figure in creating the active aging concept but also actively standing behind some of the initiatives of the age-friendly cities that are described in the second part of the book. The chapters in the first part are a very valuable source of information on the active aging concept and on how it was later operationalised by the age-friendly cities movement. The authors also present some controversies that emerged around the discussion on age-friendliness. For example, Alan Walker draws attention to the fact that “age-friendly” often refers to the “old age-friendly” instead of “aging-friendly.” In this way when thinking of age-friendly solutions, they are too often focused on just the needs of older adults and not the needs of people throughout their whole life course. The use of checklists of age-friendly cities domains is also critically discussed. It is being pointed out that they have not been used entirely in the same way by various cities or communities which instead can adopt different diagnostic approaches. This flexibility is a great value of the AFCC network which includes cities and communities from various parts of the world.

The second part of the book focuses on the implementation of the AFCC idea. It presents nine experimental AFCC programs which refer to various locations worldwide and are in different scales, ranging from a city and community level, through national programs to international developments. The chapters present examples from Australia, South and North America, Western Europe, and China (Hong Kong). It remains unanswered why such a selection of examples was provided. It is being remarked that Africa was excluded. However, other areas were not elaborated in-depth as well. For example, it would be prudent to complete the presented examples by at least one from the former Soviet bloc. The countries from this area face a much quicker aging process than the Western ones, and it would be highly interesting also to learn from their experiences. It is essential because—as the authors of the Hong Kong chapter remark—the guidelines and checklists for age-friendly cities are so far mainly dedicated to cities and communities in developed countries. It is vital for the future development of the GNAFCC to adopt different approaches that would also fit other parts of the world.

Although the presented initiatives differ a lot (both in scale and location), they have one thing in common. Those which managed to be very successful (e.g., the case study of New York City, USA) were from the start supported by the authorities. Those cases of initiatives where such support was missing, despite great engagement of older people and scholars, did not succeed that much. Therefore, the role of the authorities both on local and state level is significant in creating effective age-friendly policies. Equally important is also know-how and general commitment of city/community officials to introduce the age-friendly concepts as the example of region Wallonia, Belgium proves. Growing know-how on age-friendly environments is increasingly important. This issue was underlined by Anne-Sophie Parent and Julia Wadoux from Age Platform Europe, a network that is highly active in promoting the idea of age-friendly environments among the European policymakers on all policy levels.

The editors did not force the authors of the chapters to follow the same evaluation rules in the described programs as they argued that providing a one evaluation model is one of the main difficulties regarding the AFCC research field. It is understandable when referring to presented national or international programs. However, cities examples should have the same content structure that could help to understand the peculiarities of a given case study better.

The third part of the book discusses future and current challenges of the AFCC, including the issue of “aging active” vs. “healthy aging” that seems to be the most pronounced. The stress on active aging is evident. While the authors of the described papers still value active aging, nowadays it is rather healthy aging which seems to be a more critical approach. Age-friendly movement is considered to be so far overly focused on active aging which, for example, excludes interventions toward people with dementia or physical disability. Thus, nowadays healthy aging with health promotion and early detection of health risk conditions is being supported.

Rapid growth in the number of GNAFCC members and a transition from active aging to healthy aging is a proof for a great dynamic of the discussion. Despite this fact, the book is a highly recommendable publication. The contributions referring to the origins AFCC give valuable insights. The others show state of the art for the year 2015 and are also an essential source of information on how the age-friendly movement emerged. AFCC movement flourishes in areas where experts in this field, dedicated officials, and policymakers are present. That is why it is a must-read for every scholar investigating the age-friendly movement. Although this book rather cannot be regarded as a handbook, it is recommendable for all dealing with the AFCC topic.

## Author Contributions

The author confirms being the sole contributor of this work and has approved it for publication.

### Conflict of Interest Statement

The author declares that the research was conducted in the absence of any commercial or financial relationships that could be construed as a potential conflict of interest.

